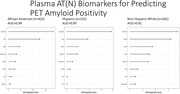# Examining the utility of plasma Alzheimer’s disease biomarkers across diverse populations

**DOI:** 10.1002/alz.086631

**Published:** 2025-01-09

**Authors:** Sid E. O'Bryant, Melissa Petersen

**Affiliations:** ^1^ University of North Texas Health Science Center, Fort Worth, TX USA

## Abstract

**Background:**

Plasma biomarkers of Alzheimer's disease were examined in the Health & Aging Brain Study ‐ Health Disparities (HABS‐HD). Data from n>3,000 participants (n>1,000 African American, n>1,000 Hispanic, n>1,000 non‐Hispanic white) were examined in relation to cognitive diagnoses, functional outcomes and imaging (MRI, PET amyloid and PET tau) outcomes.

**Method:**

Plasma biomarkers were examined via single molecule assays using the Simoa technology. MRI was conducted using 3T Siemens scanner using ADNI protocols. PET amyloid (Neuraceq) and PET tau (PI2620) were captured using Siemens PET/CT scanners. All imaging data was examined using standardized pipelines at LONI.

**Result:**

Plasma biomarkers of amyloid, tau and neurodegeneration vary by race/ethnicity. Plasma proteomic profiles were highly accurate in detecting MCI/dementia as well as amyloid positivity across race/ethnic groups; however, the protein importance plots varied across groups. Plasma biomarkers were also significantly associated with functional outcomes. Finally, measures of metabolic functioning were also associated with amyloid and tau outcomes, which varied by race/ethnic groups.

**Conclusion:**

Plasma biomarkers can be used in multi‐step screening procedures for increasing inclusion in clinical research. Additionally, these biomarkers vary with regards to level and clinical impact across race and ethnic groups. Therefore, additional work is needed to understand plasma biomarkers across diverse populations in order to establish them as front‐line rule out screening tools.